# Premature Ventricular Contractions From the Proximal Left Anterior Fascicle: Insight From the Electrophysiologic and Anatomic Parameters

**DOI:** 10.1111/jce.70164

**Published:** 2025-10-27

**Authors:** Xuan Hoang Le, Chin Yu Lin, Fa Po Chung, Yenn Jiang Lin, Shih Lin Chang, Li Wei Lo, Yu Feng Hu, Ta Chuan Tuan, Tze Fan Chao, Jo Nan Liao, Guan Yi Li, Ting Yung Chang, Ling Kuo, Cheng I. Wu, Chih Min Liu, Shin Huei Liu, Ming Jen Kuo, Yu‐Shan Huang, Lo Chieh Ling, Cheng Han Chan, Da Wei Chang, Minh Nhut Nguyen, Paula Victoria Catherine Cheng Bromeo, Shih Ann Chen

**Affiliations:** ^1^ Heart Rhythm Center and Cardiovascular Center, Taipei Veterans General Hospital Taipei Taiwan; ^2^ Interventional Cardiology Center, Tam Anh General Hospital Ho Chi Minh Vietnam; ^3^ Department of Medicine National Yang‐Ming Chiao‐Tung University Taipei Taiwan; ^4^ Heart Rhythm Center, Division of Cardiology, Department of Medicine Taichung Veterans General Hospital Taichung Taiwan; ^5^ Division of Cardiology, Department of Medicine Tri‐Service General Hospital Taipei Taiwan; ^6^ National Chung Hsing University Taichung Taiwan; ^7^ Department of Medicine China Medical University College of Medicine Taichung Taiwan

**Keywords:** ablation, fascicle reverse, premature ventricular contraction, proximal left anterior fascicle, right coronary cusp

## Abstract

**Introduction:**

Premature ventricular contractions (PVCs) originating from the proximal left anterior fascicle (LAF) are rarely discussed. We aimed to describe the characteristics and ablation outcome of PVCs originating from the proximal LAF and propose a stepwise approach.

**Method and Results:**

From 2018 to 2024, 16 patients (nine males) with proximal LAF–PVCs who underwent ablation were enrolled, and their electrophysiological characteristics and procedural details were analyzed. The acute procedural outcomes were complete success, success with residual nonclinical PVCs, and partial success (decrease in clinical PVCs). The electrocardiographs of the PVCs showed a right bundle branch block with an inferior axis. In the initial ablation attempt, targeting the earliest left ventricular (LV) endocardial activation site eliminated clinical PVC in 14 patients. Nonclinical PVC occurred in seven patients. A second attempt from the right coronary cusp (RCC) was made in four patients (three nonclinical and one clinical PVC), which resulted in complete success. One patient (6.3%, partial success) had PVC recurrence. Sites with a successful ablation showed a significantly greater Fascicle‐QRS[PVC] interval, Delta Fascicle‐QRS[PVC–Sinus] interval, and more reverse fascicle potentials than other sites. Patients who underwent successful supra‐RCC ablation had a shorter RCC‐LV earliest activation site distance (EAS).

**Conclusion:**

This is the first study to investigate the dynamic electrogram property in LAF–PVC ablation. The Fascicle–PVC interval and reverse fascicle are useful for predicting acute ablation success. The RCC approach may be effective in cases with a short RCC–LV EAS distance. Nonclinical PVC morphology could be due to LAF block and might not affect the long‐term outcome after careful mapping.

## 1 Introduction

Premature ventricular complexes (PVCs) and ventricular tachycardias (VTs) originating from the His‐Purkinje system are increasingly being recognized as substrates for catheter ablation [1]. The left posterior fascicle (LPF) is the most common origin of arrhythmia within the left fascicular system. Arrhythmias arising from the left anterior fascicle (LAF) are less frequent [2]. Unlike other idiopathic PVCs [3], which may arise from various ventricular locations, LAF–PVCs exhibit distinctive electrocardiographic (ECG) characteristics, often presenting with a right bundle branch block (RBBB) morphology and an inferior axis. These unique features can complicate both diagnosis and treatment, making the management of LAF–PVCs particularly challenging.

Within the LAF arrhythmia spectrum, those originating from the proximal segment represent a distinct subgroup with unique considerations for ablation. The definition of “proximal” LAF consistently relates its origin to the initial portion of the fascicle, near the common trunk of the left bundle branch (LBB), and anatomically situated beneath the right coronary cusp (RCC) of the aortic valve [4]. This specific anatomical relationship, where the proximal LAF lies immediately adjacent to the RCC, forms the basis for alternative ablation strategies targeting the RCC instead of the left ventricular (LV) endocardium [5]. This proximity to both the RCC and the central conduction system (His bundle, proximal LBB) presents both challenges and therapeutic opportunities.

Catheter ablation is a crucial therapeutic option for patients with symptomatic, drug‐refractory PVCs. The intricate anatomy of the LV conduction system and the proximity of the LAF to critical structures contribute to procedural complexity [6]. Additionally, the occurrence of transient or permanent conduction disturbances, such as left anterior fascicular block (LAFB), raises further concerns regarding procedural safety [7].

Recently, advancements in electrophysiological mapping and catheter‐based ablation techniques have improved the understanding and management of fascicular PVCs. Additionally, the role of alternative ablation sites, including the supra‐RCC approach, has gained increasing attention as a means of achieving procedural success in challenging cases.

In this study, we aimed to comprehensively characterize PVCs originating from the proximal LAF and evaluate the outcomes of catheter ablation to contribute valuable insights into the electroanatomic features, procedural strategies, and clinical management of this rare arrhythmia, ultimately informing clinical decision‐making and optimizing patient care.

## 2 Methods

### 2.1 Study Population

**Figure 1:**
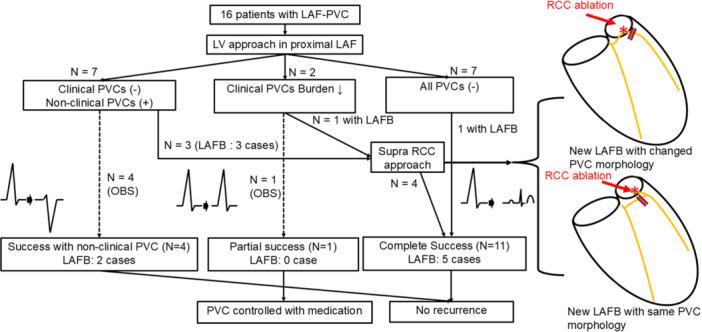
Study flowchart. This figure summarizes the stepwise approach for all patients.

Between January 2018 and January 2024, 16 patients diagnosed with LAF–PVC without structural heart disease were included in this study. LAF–PVC was characterized by PVC–QRS with a marked inferior frontal plane QRS axis (qR in the inferior leads and rS in leads I and aVL), along with either typical or atypical RBBB. All patients underwent 12‐lead ECG, 24‐h Holter monitoring, transthoracic ECG, coronary arteriography, magnetic resonance imaging, and electrophysiological evaluation. This retrospective study was approved by the Institutional Review Board. The research reported in this paper adhered to the Helsinki Declaration guidelines. A flowchart of the study population is presented in Figure 1.

### 2.2 Electrophysiological Study

All antiarrhythmic drugs were discontinued for at least five half‐lives before the procedure [8]. The electrophysiological study was performed after an overnight fast, with mild sedation. During the procedure, the LV mapping was performed using a retrograde approach via the left femoral artery for mapping and ablation, utilizing a three‐dimensional (3D) electroanatomic mapping system (EnSite Precision System, Abbott Medical Inc., St. Paul, MN, USA, or CARTO System, Biosense‐Webster Inc., Diamond Bar, CA, USA). Before introducing the catheter into the left ventricle, 3D anatomical reconstruction and mapping of the aortic root, specifically focusing on the three coronary cusps, were performed. Intracardiac electrograms were recorded with a digital electrophysiological recording system (LabSystem, Bard Electrophysiology, Lowell, MA, USA), filtered between 30 and 300 Hz.

A linear multielectrode mapping catheter was used (DECANAV mapping catheter or Livewire Steerable Electrophysiology Catheter) to identify the Purkinje potentials. Detailed activation mapping was conducted to identify the earliest activation site (EAS) of PVCs, particularly a sharp, high‐frequency, presystolic Purkinje potential preceding the PVC‐QRS complex at the RCC and left ventricle. If LAF–PVC was absent at baseline, an isoproterenol infusion (1–4 mg/min) was administered to increase the heart rate by 20%.

Radiofrequency ablation was performed using a 3.5‐mm open‐irrigated ablation catheter (Thermocool, Biosense Webster Inc; FlexAbility, Abbott), with energy delivered at the earliest Purkinje potential during PVC. If the PVCs were suppressed in the initial 20 s, the energy application would be continued for three lesions. If the PVCs were not suppressed after 20 s, the location of the ablation catheter was adjusted. Before ablation near the RCC, angiography was performed to verify the position of the ablation catheter, the coronary arteries, and to assess ablation safety. The power output was adjusted from 25 to 35 W, with a maximum target temperature of 40°C for 3–5 lesions.

When the PVC morphology changed, a detailed mapping was repeated to identify the EAS. Under Isoproterenol infusion and during 30‐min observation postablation, the acute procedural outcomes were defined as: complete success (no PVC left), success with residual nonclinical PVC, and partial success (decrease in clinical PVC burden).

### 2.3 Analysis of the Ablation Site

The local electrograms of the ablation site before energy was applied were extracted for further analysis. If the PVC completely disappeared after ablation, the ablation was considered successful; otherwise, it was considered unsuccessful. We measure the following parameters (Supporting Information S1: Figure 1): (1) fascicle to QRS during SR and PVC, (2) the difference between fascicle to QRS during SR and PVC, and (3) whether fascicle signals reverse or not (the change in the first spike on the bipolar electrode).

### 2.4 Follow Up

Surface ECGs and 24‐h Holter recordings were routinely conducted after the procedure, as well as at 3‐ and 6‐months following discharge. Additionally, an ECG and 24‐h monitoring were performed whenever patients experienced palpitations [8, 9].

### 2.5 Statistical Analysis

Data were analyzed using SPSS 18.0. Continuous variables are presented as the mean ± SD. Group differences for continuous data were assessed using the Mann–Whitney two‐sample test. Categorical variables were compared using the *χ* ^2^ test. A *p* value of < 0.05 was considered statistically significant.

## 3 Results

### 3.1 Patient Characteristics

Overall, 16 patients (nine males, mean age 44.5 ±17.2 years old) were enrolled in this study. The mean PVC burden and left ventricular ejection fraction (LVEF) were 18.2 ± 10.4% and 57.4 ± 6.3%, respectively. The baseline characteristics are summarized in Table 1. Two patients underwent catheter ablation before this procedure. One patient received ablation for PVC originating from the left posterior fascicle. The other patient underwent ablation for LAF–PVC complicated with complete AV block in other hospitals.

**Table 1:** Baseline characteristics of patients.

Patient	Age	Gender	Comorbidity	Medication history	PVC burden	LVEF
No. 1	26	M	—	Diltiazem	11.4%	59%
No. 2	31	F	—	Diltiazem	12.7%	52%
No. 3	52	F	DM, CAD, HT	Beta‐blocker	13.6%	61%
No. 4	55	F	CAD	Beta‐blocker	21.0%	38%
No. 5	24	M	—	Beta‐blocker	46.0%	51%
No. 6	19	M	—	Diltiazem, Propafenone	10.0%	60%
No. 7	39	M	—	Fleicainde, Propafenone	15.0%	61%
No. 8	29	M	—	Fleicainde, Propafenone	9.0%	53%
No. 9	44	F	—	Flecainde	5.0%	62%
No. 10	31	F	—	Flecainde	13.2%	60%
No. 11	52	F	—	Fleicainde, Propafenone	15.0%	60%
No. 12	35	M	CAVB	None	32.0%	64%
No. 13	64	F	CAD, HT	Flecainde	9.7%	64%
No. 14	71	M	DM, HT	Flecainde	20.0%	58%
No. 15	19	M	—	Beta‐blocker	13.0%	60%
No. 16	71	M	CAD, HT	Beta‐blocker	14.0%	60%

Abbreviations: CAD, coronary artery disease; CAVB, complete AV block; DM, diabetes mellitus; F, female; HT, hypertension; LVEF, left ventricular ejection fraction; M, male; PM, Pacemaker; PVC, premature ventricular contraction; SR, sinus rhythm.

### 3.2 Mapping and Ablation

During the electrophysiological procedure, spontaneous LAF–PVCs frequently occurred in all 16 patients. The electrophysiological mapping parameters of PVCs are listed in Table 2. The EAS was confirmed at the left ventricle in all patients with LAF–PVC, with a presystolic Purkinje potential (i.e., fascicular potential) that preceded the PVC‐QRS by 32.88 ± 9.31 ms and preceded the SR‐QRS by 27.60 ± 8.37 ms (delta of fascicle to QRS = 4.47 ± 10.3) (Table 2). Ablation at these sites successfully achieved target PVC elimination in 14 patients. In one patient, the same PVC morphology recurred 10 min after ablation, and further ablation with half‐saline achieved a durable success.

**Table 2:** Details of the procedural data.

Case	Fascicle‐QRS (SR in LV), ms	Fascicle‐QRS (PVC in LV), ms	Reversal of fascicle (LV)	successful with first site in LV	Second ablation	Fascicle‐QRS (SR in RCC), ms	Fascicle‐QRS (PVC in RCC), ms	Reversal of fascicle (RCC)	PVC changes morphology	LAFB after ablation	Acute result	Follow up
No. 1	−36	−36	y	n	RCC	−24	−45	y	y	y	Complete success	No recur
No. 2	−24	−24	n	y					y	y	Success with nonclinical PVC	No recur
No. 3	−34	−34	n	n	RCC	−38	−66	y	y	y	Complete success	No recur
No. 4	−38	−38	n	n	RCC	−18	−38	y	y	y	Complete success	No recur
No. 5	−18	−18	y	y					n		Success with nonclinical PVC	No recur
No. 6	−28	−30	y	y					n	y	Success with nonclinical PVC	No recur
No. 7	−34	−54	n	y					n		Complete success	No recur
No. 8	−26	−10	n	y					y		Partial success	recur
No. 9	−24	−26	y	n	RCC	−30	−42	y	n	y	Complete success	No recur
No. 10	−37	−42	n	y					n		Complete success	No recur
No. 11	−39	−38	y	y					n		Complete success	No recur
No. 12	n/a	−45	n/a	y					n	y	Complete success	No recur
No. 13	−26	−32	y	y					y		Complete success	No recur
No. 14	−8	−34	y	y					n		Success with nonclinical PVC	No recur
No.15	−15	−34	y	y					n		Complete success	No recur
No.16	−27	−31	n	y					n		Complete success	No recur

Abbreviations: Fascicle‐QRS (PVC in LV), Fascicle‐QRS interval of PVC recorded at left ventricle EAS; Fascicle‐QRS (SR in LV), Fascicle‐QRS interval of sinus rhythm recorded at left ventricle EAS; Fascicle‐QRS (PVC in RCC), Fascicle‐QRS interval of PVC recorded at supra right coronary cusp EAS; Fascicle‐QRS (SR in RCC), Fascicle‐QRS interval of sinus rhythm recorded at supra right coronary cusp EAS; LV, left ventricle; n, no; Reversal of fascicle, defined as the reversal in the direction of fascicular electrical activation; SupraRCC, Supra right coronary cusp; y, yes.

**Figure 2:**
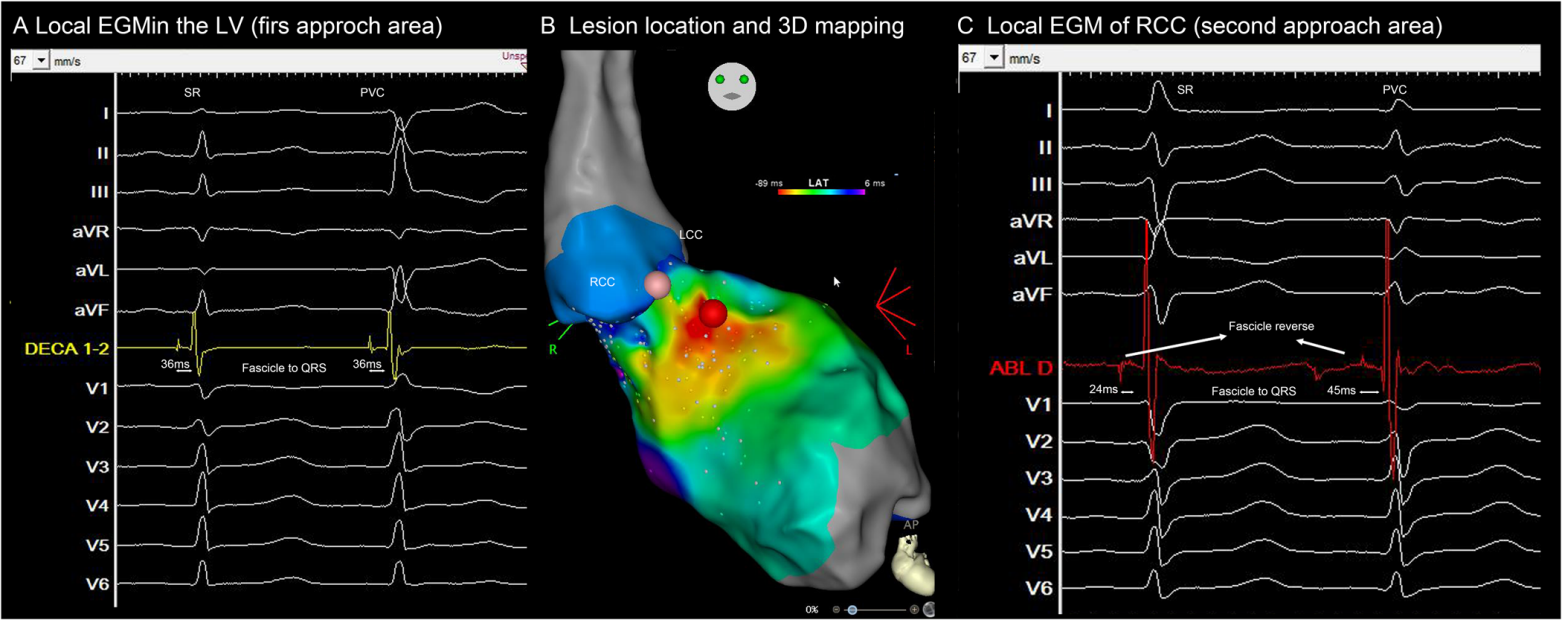
Example of a case with final successful ablation from RCC. This figure illustrates a change in PVC morphology after the first ablation attempt in the LV proximal LAF area. Re‐mapping of the new PVC showed an early fascicular potential in the RCC area. Both areas showed reverse fascicular potentials. (A) Before the first attempt in the LV, the fascicle‐to‐QRS was 36 ms for both SR and PVC beats. The Delta Fascicle‐QRS[PVC–Sinus] was 0. (B) Activation mapping identified the earliest activation area in the LV endocardium, and the second approach was located at the RCC, above the cusp. (C) After the first attempt, the second attempt in the RCC demonstrated fascicular potentials. In this area, the fascicle‐to‐QRS was 24 ms during SR and 45 ms during PVC beats. The Delta Fascicle‐QRS [PVC–Sinus] was 21 ms.LAF, left anterior fascicle; LV, left ventricle; PVC, premature ventricular complexes, RCC, right coronary cusp.

In two patients, the clinical PVC persisted after the initial ablation. Re‐mapping of the LAF, supra‐RCC, and LPF was performed. Seven other patients developed nonclinical PVCs during the observation period, for which supra‐RCC and LPF were mapped. Early fascicular signals originating from the RCC area were identified in four of these patients (three with nonclinical PVCs and one with a clinical PVC). These four patients underwent a second ablation attempt from the supra‐RCC, which achieved complete success without any residual PVCs (Table 2 and Figure 2).

The four patients who received RCC ablation demonstrated LAFB after LV endocardial ablation. The successful ablation site of RCC could be in the upper stream of the blocked fascicle in three patients and the distal branch of LAF in one patient (Figure 1). In the other four patients with nonclinical PVCs, the PVC burden decreased during mapping, and no further energy was applied. In one patient with decreased burden of clinical PVCs, further ablation was performed due to transient atrioventricular block during the procedure.

### 3.3 Characteristics of Local Electrograms in the Ablation Site (Table 3)

**Table 3:** Characteristic of ablation point.

	Successful site (*n* = 11 or 10*)	Site without complete success (*n* = 11)	*p* value
Fascicle potential preceded* SR‐QRS, ms	27.90 ± 9.33	28.73 ± 6.71	0.820
Fascicle potential preceded PVC‐QRS, ms	45.45 ± 12.37	27.81 ± 58 8.69	0.001
Delta Purkinje to QRS (PVC – SR), ms	19.30 ± 7.33	−0.91 ± 5.39	< 0.001
Reversal fascicle signal during SR and PVC*	9 (90%)	4 (36.4%)	0.025

Abbreviations: CAVB, complete atrioventricular block; PVC, premature ventricular complex; SR, sinus rhythm.

* Data from case No. 12 (single ablation in the LV eliminated the PVC) was not available due to CAVB.

A total of 11 sites with successful ablation (elimination of all PVC) and 11 sites with unsuccessful ablation (partial success or residual nonclinical PVC) were recorded. Sites with successful ablation showed a significantly greater Fascicle‐QRS [PVC] interval (45.45 ± 12.37 vs. 27.81 ± 8.69, *p* = 0.011), Delta Fascicle‐QRS [PVC–SR] interval (19.30 ± 7.33 vs. −0.91 ± 5.39, *p* < 0.001), and more reverse fascicle (9 (90%) vs.4 (36.4%), *p* = 0.025).

**Figure 3:**
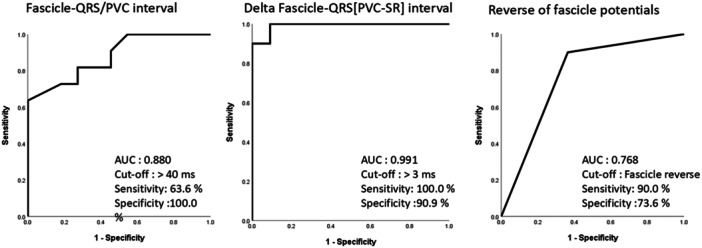
Prediction of the successful site. Receiver operator characteristic (ROC) curve for fascicle to QRS, delta fascicle to QRS (PVC‐SR), and reverse fascicle potentials in predicting the successful site.

Characteristics of sites with and without a complete ablation are presented in Table 3. Figure 3 shows the sensitivity and specificity of Fascicle‐QRS [PVC] interval, Delta Fascicle‐QRS [PVC–SR] interval, and the reverse fascicle in predicting the site with successful ablation. The best cut‐off value to Fascicle‐QRS [PVC] and Delta Fascicle‐QRS [PVC–SR] interval is 40 ms and 3 ms, respectively.

Patients who experienced failed LV endocardial ablation but had successful supra‐RCC ablation had a shorter RCC‐LV EAS distance compared with patients with successful LV ablation (20.00 ± 3.92 mm vs. 30.13 ± 14.34 mm, *p* < 0.01). LAFB postablation was observed in seven of 15 patients, but did not affect the outcome (*p *= 0.55). The change in QRS axis is summarized in the Supporting Information S1: Table 1.

### 3.4 ECG Characteristics

**Figure 4:**
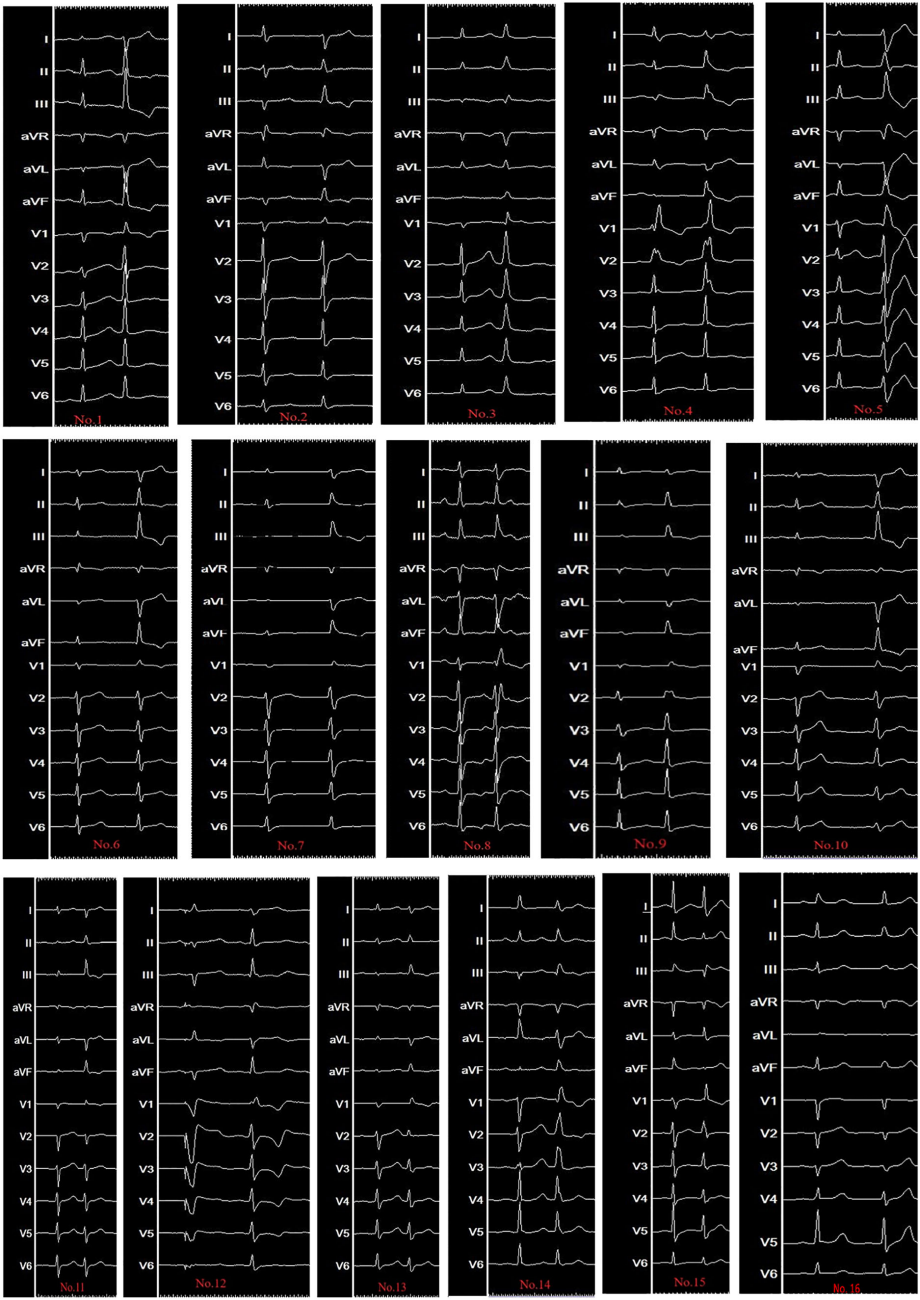
Surface electrocardiography of all 16 patients. This figure summarizes the baseline rhythm and the PVC beats.

SR QRS mean duration was 86.9 ± 15.9 ms, while PVC QRS mean duration was 107.8 ± 18.1 ms. The ECG morphology of baseline rhythm and PVC is listed in Figure 4. The SR morphology after ablation showed LAFB in seven of 16 patients. There was no correlation between LAFB and the rate of successful ablation (*p* = 1.00, Fisher's exact test).

During ablation, clinical PVC disappeared, and nonclinical PVC morphology appeared in seven patients. The remap of the PVC demonstrated EAS of the fascicle in the RCC in three patients. Catheter ablation applied in the RCC EAS area ameliorated the nonclinical PVC successfully. In the other four patients, the nonclinical PVC burden decreased, and no clear early fascicle potentials were found in RCC, the LAF area, or the LPF area. At the end of the procedure, four patients presented with rare residual nonclinical PVC. The residual nonclinical was not associated with clinical outcome after long‐term follow‐up with ECG monitoring.

### 3.5 Follow Up

No complications were observed in the patients during the perioperative period.

After a mean follow‐up of 14.3 ± 8.2 months following the last ablation procedure, one patient (partial success) presented with PVC recurrence 3 months after ablation. Flecainide was prescribed, and the PVC burden was less than 1% under medication. The Holter study in the other 15 patients demonstrated PVC burden less than 1% without medication.

## 4 Discussion

### 4.1 Main Findings

The primary findings suggest that the Fascicle‐QRS [PVC] interval, the Delta Fascicle‐QRS [PVC–Sinus] interval, and the reverse of fascicle potential are useful predictors of successful ablation. Additionally, the supra‐RCC approach proved effective, particularly in cases with a short RCC‐LV EAS distance. A cut‐off of 40 ms and 3 ms for Fascicle‐QRS [PVC] interval and delta Fascicle‐QRS (PVC‐SR) has a good sensitivity to predict successful ablation site, respectively. The presence of a reverse fascicle may also be an objective predictor for sites with successful ablation. The nonclinical PVC after ablation might not affect clinical outcome in patients with LAF–PVC.

### 4.2 Changes in PVC Morphology During Ablation and the Role of the RCC Approach

During the ablation procedure, changes in PVC morphology were noted in over half of the patients. While these changes may indicate tissue modification, no significant correlation was observed between morphological changes and ablation success. This finding emphasizes the importance of precise mapping and careful interpretation of electroanatomic signals to ensure procedural efficacy.

The change in PVC morphology could be due to several mechanisms:

Mechanism 1: catheter ablation blocks the distal exit of the LAF. The PVC originating from the more proximal LAF exit to the other fascicle results in a superior axis [10].

Mechanism 2:　Junctional beats after catheter ablation in the fascicle may cause different QRS morphology because the LAF was damaged [11].

Mechanism 3: Differences in PVC from other origins might present a distinct QRS morphology.

The RCC approach was performed after mapping the LPF area to exclude the possibility of a second PVC. The complete success of the RCC ablation might be explained by mechanism 1. The RCC approach could target a more proximal site of the LAF compared with the LV endocardium. In the other patient, the good long‐term result, despite the inability to find fascicular potentials for the RCC approach, could be explained by mechanism 2.

The RCC approach was a crucial alternative for patients who did not achieve success with the endocardial LVA. Shorter RCC‐LV EAS distances were associated with higher success rates when the RCC approach was used. In our study, cases with successful supra RCC approach always had an EAS in the supra RCC than in the LV endocardium, suggesting a retro‐aortic root branch origin, which was mentioned in a prior report [12, 13]. This finding highlights the importance of considering anatomical proximity and electrical activation timing when selecting the optimal ablation strategy. The ability to achieve complete success in all RCC approach cases further supports its role as a reliable alternative.

### 4.3 Risk of Atrioventricular Node Injury

In our study cohort, one patient experienced a complete atrioventricular nodal block during a prior ablation at another hospital. The ablation site was the proximal portion of the LAF in the LV endocardium. Another patient showed transient atrioventricular node injury after energy application in the proximal LAF endocardium. Neither of these two patients had an underlying cardiac disease or conduction disturbance. The atrioventricular node injury is mainly due to the procedure itself. A retrograde transaortic approach might complicate atrioventricular node injury in a rare situation [14]. A prior study also reported complications after LAF ablation from the LV, including atrioventricular block and complete LBB block in a young population (both less than 10 years old) [15]. These findings suggest that endocardial LV ablation in the proximal LAF is associated with a non‐negligible risk of atrioventricular node injury, especially in young patients receiving an endocardial ablation in the proximal LAF [2]. Supravalvular RCC approach is associated with a low risk of atrioventricular block and might be useful in patients with a short RCC‐LV EAS distance.

### 4.4 Clinical Implications

**Figure 5:**
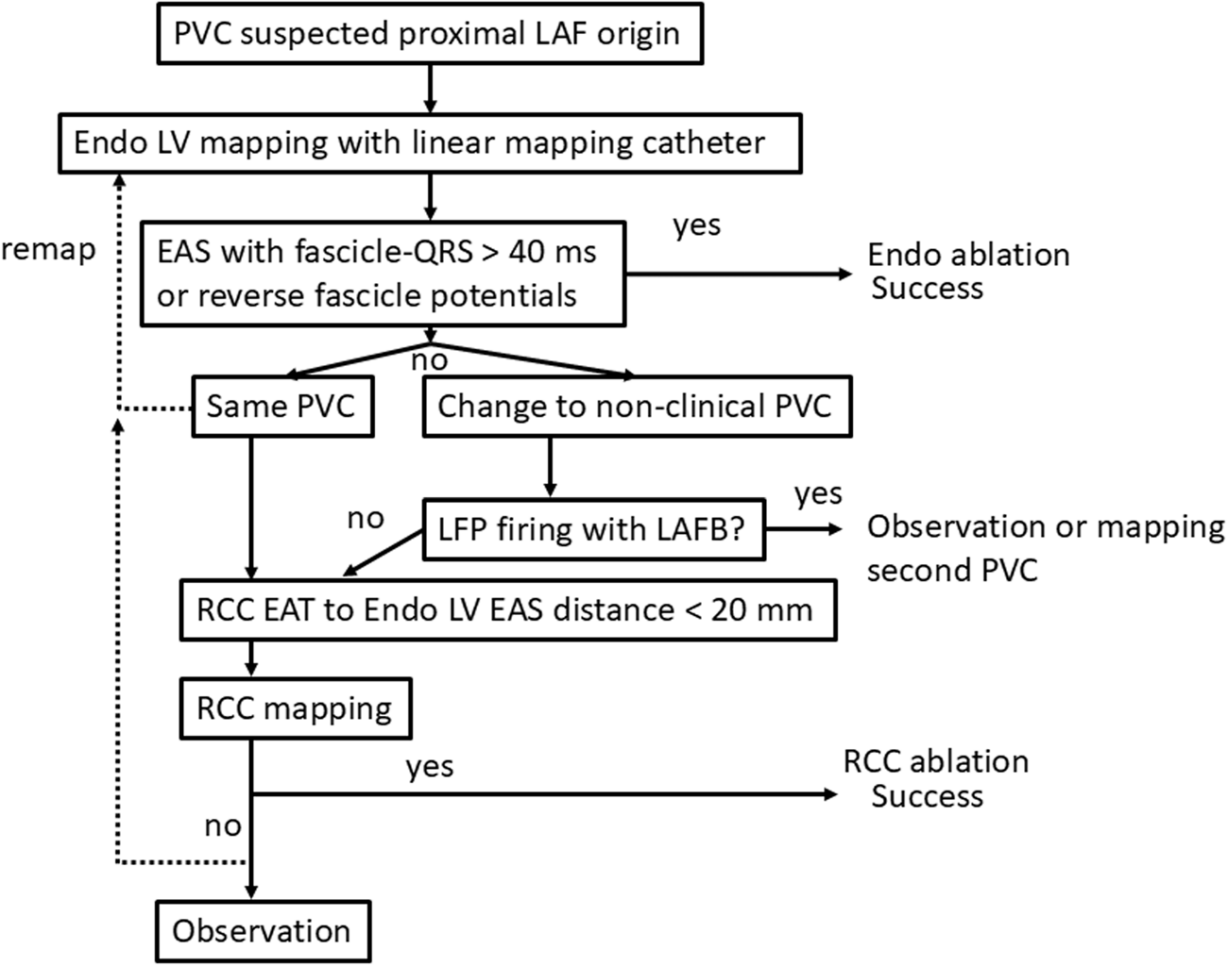
Step‐by‐step approach. This figure summarizes the proposed flowchart for the step‐by‐step approach.

The results of this study underscore the utility of the Fascicle‐QRS [PVC], Delta Fascicle‐QRS [PVC–Sinus] intervals, and reverse fascicle potential in predicting successful ablation sites. These electrophysiological parameters may serve as practical indicators during the mapping process, aiding operators in real‐time decision‐making. Additionally, the findings support the use of the RCC approach in cases with anatomical challenges or unsuccessful endocardial attempts. A simple step‐by‐step approach for LAF PVC ablation is illustrated in Figure 5.

Furthermore, the observed incidence of LAFB postablation did not significantly affect clinical outcomes. This suggests that while fascicular conduction disturbances can occur, they may not have long‐term adverse effects, reinforcing the safety of the procedure.

### 4.5 Limitation

This is a single‐center study with a limited number of cases. There was one case with a paced heartbeat, which may alter the data result compared with those of other studies. The lack of a control group limits the ability to generalize the findings. Additionally, procedural variability and operator experience may influence the results. Future multicenter, prospective studies with larger cohorts are warranted to validate these findings. Patients could not provide a precise first episode of PVC, making it impossible to find a relationship between arrhythmia history and LVEF function. The supravalvular RCC fascicular signal was not mapped before LV endocardial ablation in our study. Further studies on a systemic mapping of the supravalvular RCC and LV endocardium are warranted. A ten‐electrode linear mapping catheter was used in this study instead of a high‐resolution mapping catheter, which might have increased the fidelity of the map, especially when looking for Purkinje potentials.

## 5 Conclusion

The Fascicle‐QRS [PVC], Delta Fascicle‐QRS [PVC–Sinus] intervals, and reverse fascicle potentials are valuable predictors of ablation success in patients with LAF‐PVCs. The supra‐RCC approach offers an effective alternative when endocardial ablation is unsuccessful, especially in patients with a short distance between the RCC EAS and LV EAS. Residual nonclinical PVC should be carefully mapped because the LAFB might change the LAF–PVC exit proximal to the initial ablation site. After careful mapping, a residual nonclinical PVC may not be associated with long‐term outcomes. This finding might be essential for the clinical management of LAF–PVCs.

## Ethics Statement

The studies involving human participants were reviewed and approved by the local institutional review board. Written informed consent to participate in this study was provided by the participants' legal guardian/next of kin.

## Conflicts of Interest

The authors declare no conflicts of interest.

## Supporting information

Supplemental file revise 0925.

## Data Availability

The original contributions presented in the study are included in the article/Supporting Information S1. Further inquiries can be directed at the corresponding author.
