# Supplementary material for: Premature Ventricular Contractions From the Proximal Left Anterior Fascicle: Insight From the Electrophysiologic and Anatomic Parameters
Source: J Cardiovasc Electrophysiol. 2025 Oct 27;37(1):7–16. doi: 10.1111/jce.70164 (PMC12794799; doi:10.1111/jce.70164)
Supplement: Supplementary file 1 — Supplemental file revise 0925. [file JCE-37-7-s001.docx]

| Supplemental Table 1 | | | | | | | | | | |
| --- | --- | --- | --- | --- | --- | --- | --- | --- | --- | --- |
|  | baseline rhythm | QRS duration pre ablation, ms | QRS axis pre ablation | QRS duration post ablation, ms | LAF block post ablation | QRS axis post ablation | PVC duration, ms | BCL, ms | CI, ms |  |
| No.1 | SR + PVC | 78 | 65 | 90 | Y | -21 | 81 | 741 | 540 |  |
| No.2 | SR + PVC | 94 | -59 | 102 | Y | -44 | 94 | 698 | 601 |  |
| No.3 | SR + PVC | 88 | 21 | 104 | Y | -61 | 100 | 769 | 460 |  |
| No.4 | SR (RBBB) + PVC | 134 | 16 | 138 | Y | -55 | 144 | 750 | 453 |  |
| No.5 | SR + PVC | 84 | 71 | 82 |  | 113 | 118 | 528 | 440 |  |
| No.6 | SR + PVC | 78 | 107 | 84 | Y | -36 | 100 | 1066 | 710 |  |
| No.7 | SR + PVC | 80 | 42 | 97 |  | 20 | 113 | 741 | 545 |  |
| No.8 | SR + PVC | 102 | 95 |  |  | -3 | 104 | 552 | 396 |  |
| No.9 | SR + PVC | 100 | 29 | 110 | Y | -42 | 100 | 824 | 440 |  |
| No.10 | SR + PVC | 98 | 38 | 92 |  | 60 | 128 | 1000 | 598 |  |
| No.11 | SR + PVC | 82 | 71 | 88 |  | 74 | 90 | 702 | 430 |  |
| No.12 | BAVIII/DDDR | 125 | 95 | 125 |  | 93 | 132 | 750 | 453 |  |
| No.13 | SR + PVC | 98 | 7 | 108 |  | -1 | 108 | 874 | 384 |  |
| No.14 | SR + PVC | 84 | 16 | 88 | Y | 4 | 135 | 864 | 408 |  |
| No.15 | SR + PVC | 96 | 55 | 98 |  | 72 | 96 | 476 | 400 |  |
| No.16 | SR + PVC | 80 | 50 | 80 |  | 46 | 83 | 864 | 516 |  |

BAVIII, 3^rd^ degree AV block; BCL, basic cycle length; CI, coupling interval; DDDR, dual-chamber pace maker with DDDR mode; LAF, left anterior fascicular; PVC, premature ventricular contraction; RBBB, right bundle branch block; SR, sinus rhythm; Y, yes

**Supplemental Figure 1. Illustration for the measurement of the fascicle to QRS and the reverse of the fascicle**


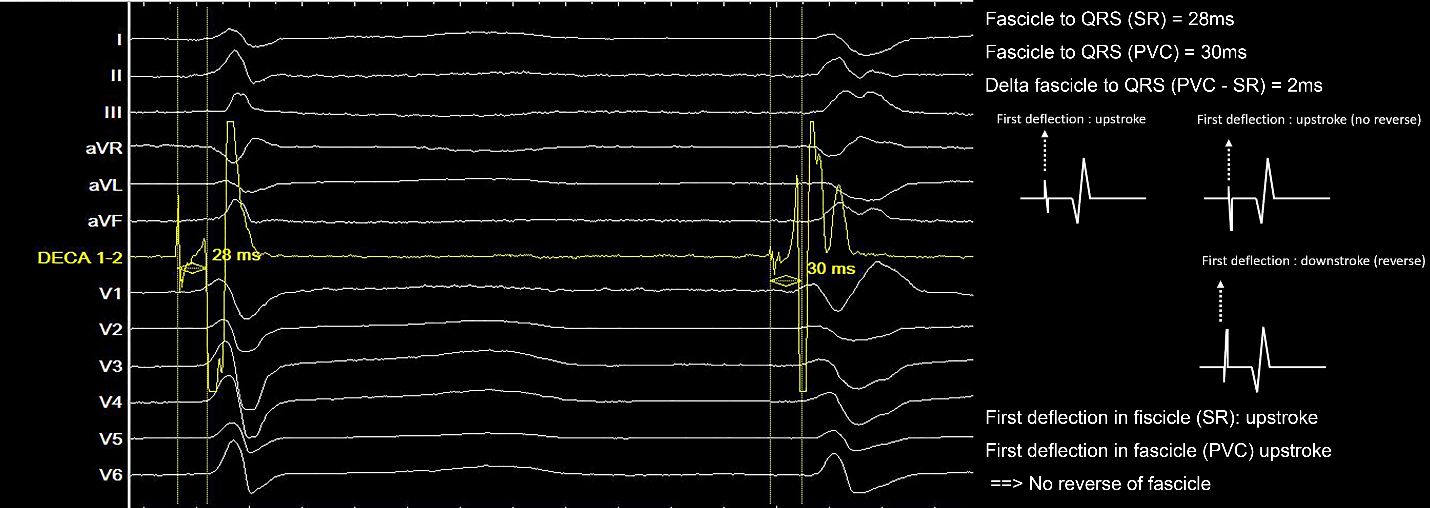


The left panel shows intracardiac electrograms in the LAF area during SR and PVC beats. The time from the fascicle potential to the QRS onset was measured with the tools in the Bard Recording system. The measurement of the delta fascicle to QRS (PVC–SR) was shown in the right panel. The reversal of fascicle potentials was defined as the change of the first deflection (upstroke or downstroke).

LAF = left anterior fascicle; SR = sinus rhythm; PVC = premature ventricular complexes
